# A novel histopathological scoring system to distinguish urticarial vasculitis from chronic spontaneous urticaria

**DOI:** 10.1002/clt2.12031

**Published:** 2021-04-28

**Authors:** Viktoria Puhl, Hanna Bonnekoh, Jörg Scheffel, Tomasz Hawro, Karsten Weller, Peter von den Driesch, Hans‐Joachim Röwert‐Huber, José Cardoso, Margarida Gonçalo, Marcus Maurer, Karoline Krause

**Affiliations:** ^1^ Dermatological Allergology Allergie‐Centrum‐Charité Department of Dermatology and Allergy Charité – Universitätsmedizin Berlin Berlin Germany; ^2^ Department of Dermatology Klinikum Stuttgart Stuttgart Germany; ^3^ Department of Dermatology and Venereology University Hospital and Faculty of Medicine University of Coimbra Coimbra Portugal

**Keywords:** chronische sponstane urtikaria, endothelzellschwellung, erythrozyten‐extravasate, fibrin, histopathologie, leukozytoklasie, urtikariavaskulitis

## Abstract

**Background:**

Urticarial vasculitis (UV) is defined by long‐lasting urticarial lesions combined with the histopathologic findings of leukocytoclastic vasculitis. As one of the major unmet needs in UV, diagnostic criteria are rather vague and not standardized. Moreover, there seems to be considerable overlap with chronic spontaneous urticaria (CSU), particularly for the normocomplementemic variant of UV. Therefore, this study aimed to develop a diagnostic scoring system that improves the histopathologic discrimination between UV and CSU.

**Methods:**

Lesional skin sections of patients with clinical and histopathologic diagnosis of UV (*n* = 46) and CSU (*n* = 51) were analyzed (blinded to the diagnosis) for the following pre‐defined criteria: presence of leukocytoclasia, erythrocyte extravasation, fibrin deposits, endothelial cell swelling, ectatic vessels, blurred vessel borders, dermal edema, intravascular neutrophil, and eosinophil numbers and numbers of dermal neutrophils, macrophages and mast cells.

**Results:**

The greatest differences between UV and CSU samples were observed for leukocytoclasia (present in 76% of UV vs. 3.9% of CSU samples; *p* < 0.0001), erythrocyte extravasation (present in 41.3% of UV vs. 2.0% of CSU samples; *p* < 0.0001), and fibrin deposits (present in 27.9% of UV vessels vs. 9.7% of CSU vessels; *p* < 0.0001). Based on these findings, we developed a diagnostic score, the urticarial vasculitis score (UVS), which correctly assigned 37 of 46 cases of UV and 49 of 51 cases of CSU to the previously established diagnosis.

**Conclusion:**

Our results suggest that the UVS, a combined quantitative assessment of the three criteria leukocytoclasia, fibrin deposits and extravasated erythrocytes, distinguishes UV from CSU in skin histopathology. The UVS, if validated in larger patient samples, may help to improve the diagnostic approach to UV.

## 1 INTRODUCTION

Urticarial vasculitis (UV) is a rare chronic and debilitating disease defined by long lasting urticarial lesions (>24 h) and histopathological findings of leukocytoclastic vasculitis.^1^ UV skin lesions come with burning or pain rather than pruritus and often resolve with purpura or hyperpigmentation.^2^ The clinical spectrum of UV shows high intraindividual and interindividual variations.^3^, ^4^, ^5^, ^6^ Systemic manifestations, such as joint involvement with arthralgia and joint stiffness, are common; and also pulmonary, gastrointestinal and renal involvement may occur.^7^, ^8^ Those symptoms are commonly associated with hypocomplementemic UV, a rare variant of UV, which is most often associated with anti‐C1q autoantibodies.^3^, ^9^ The prevalence of hypocomplementemic UV in UV patients was reported to range between 9% and 21%.^3^, ^10^, ^11^ Some cases are linked to immune‐mediated diseases such as lupus erythematosus and Sjögren's syndrome or chronic infections (e.g., hepatitis B/C, Epstein‐Barr‐virus and borreliosis), but in the majority of UV patients no underlying disease is identified.^1^, ^12^

One of the major challenges in UV, especially in its normocomplementemic variant, is the difficulty to distinguish it from chronic spontaneous urticaria (CSU).^13^ CSU presents characteristically with recurrent itching wheals with a duration >24 h, with a disease course longer than 6 weeks.^14^ Clinically, there is considerable overlap between the two diseases. Both can come with recurrent wheals and angioedema. Wheals that last longer than a day and leave transient purpura and changes in skin pigmentation upon remission are seen as signs that point to UV.^15^ Moreover, CSU also sometimes shows this phenotype, especially in cases of high disease activity.^5^, ^16^ As a result, misdiagnosis and delay in diagnosis is common in UV.^6^, ^16^, ^17^ This results in inadequate and inefficient treatment, as the first and second line treatment of CSU, standard and higher than standard doses of antihistamines, are usually not effective in UV.^15^ Therefore, early diagnosis is crucial for patients with UV, and the current guideline for managing CSU recommends, when UV is suspected, performing a skin biopsy to confirm the diagnosis.^18^

The histopathological evaluation of UV relies on a constellation of features including leukocytoclasia, erythrocyte extravasation, fibrin deposits, an inflammatory infiltrate of either neutrophils or lymphocytes and endothelial cell swelling.^3^, ^4^, ^5^, ^6^, ^8^, ^10^, ^12^, ^16^, ^17^, ^19^, ^20^, ^21^, ^22^, ^23^ However, as of now, there is no consensus on the importance of single histopathologic features for establishing the diagnosis of UV based on its histopathology.^5^, ^16^ In fact, many cases that clinically fit UV do not show overt vasculitis on histopathology^5^ and there are no validated criteria for diagnosing UV.

Tools are needed to improve the differentiation between UV and CSU and to reduce the rate of misdiagnoses and delay in diagnosis of UV. This study aimed to develop such a tool, a diagnostic histopathologic score, by combining and quantifying a set of pre‐defined histopathologic criteria.

## 2 METHODS

### 2.1 Patients and patient samples

Skin punch and spindle biopsies (ø 3–12 mm; total *n* = 97) for routine histology from lesional skin of patients with active UV (*n* = 46) and active CSU (*n* = 51) were collected at the Department of Dermatology, Charité – Universitätsmedizin Berlin, Germany and the Department of Dermatology, Coimbra University Hospital, Portugal between 2006 and 2019.

Patients fulfilled the following clinical and routine diagnostic criteria:

For CSU:

‐ Recurrent spontaneous pruritic wheals (with or without angioedema) for >6 weeks consistent with a clinical diagnosis of CSU.
‐ Response to approved urticaria treatment (standard‐dosed or updosed antihistamines or omalizumab).
‐ No symptoms of associated systemic disease such as arthralgia, fever attacks, hypocomplementemia.
‐ Routine histology of lesional skin consistent with urticaria showing no signs of vasculitis.

For UV patients:

‐ Recurrent spontaneous pruritic or burning wheals (with or without angioedema) for >6 weeks with longer lesional duration followed by transient purpura or hyperpigmentation.
‐ Insufficient response to standard‐dosed or up‐dosed antihistamines (persistence of moderate to severe symptoms as reported by the treating physician following at least a four weeks course of treatment).
‐ Routine histology of lesional skin consistent with UV.

The study was approved by the local ethics committees of the universities (EA4/005/15; EA4/108/18) and patients provided written and oral informed consent.

### 2.2 Routine histologic assessment

Paraffin‐embedded H.E.‐stained slides from lesional skin biopsies of UV and CSU patients (total *n* = 97) had been routinely assessed by dermatopathologists J.R.H. (*n* = 64 from Berlin) and J.C. (*n* = 33 from Coimbra) with basic knowledge of clinical patient data. For comparison, 28 of the 64 slides from Berlin (randomly assigned, including histopathologic diagnoses of UV and CSU assessed by J.R.H.) were additionally evaluated by P.v.D. and J.C. Both of them established a histopathologic diagnosis based on their expertise being blinded to the initial histopathologic assessment by J.R.H. and to any clinical information.

### 2.3 Selection of pre‐defined histologic criteria

Aiming at developing a diagnostic algorithm for UV, a set of pre‐defined histopathologic criteria was created after an extensive literature review (Table 1) and expert advice by dermatopathologists J.R.H., P.v.D. and J.C. This included the presence of the following 12 items to differentiate UV from CSU:

‐ Leukocytoclasia
‐ Erythrocyte extravasation
‐ Intravascular fibrin deposits
‐ Endothelial cell swelling
‐ Ectatic vessels
‐ Blurred vessel borders
‐ Dermal edema
‐ Number of intravascular neutrophils
‐ Number of intravascular eosinophils
‐ Dermal neutrophil numbers
‐ Dermal macrophage numbers
‐ Dermal mast cell numbers

**TABLE 1:** Histopathologic criteria of UV in different studies (*n* = 14)

	No. of studies (total)	No. of studies reporting criteria as essential
Leukocytoclasia	14	9^3^, ^4^, ^6^, ^10^, ^12^, ^17^, ^21^, ^22^, ^23^
Erythrocyte extravasation	10	6^4^, ^10^, ^17^, ^19^, ^21^, ^23^
Fibrin deposits	12	10^3^, ^4^, ^6^, ^10^, ^12^, ^16^, ^19^, ^21^, ^22^, ^23^
Neutrophilic infiltrates	12	5^10^, ^12^, ^19^, ^22^, ^23^
Endothelial cell swelling	8	3^10^, ^19^, ^21^
Dermal edema	4	0
Immunofluorescence	9	1^19^

### 2.4 Quantitative histomorphometry and planimetric analysis of pre‐defined histopathologic criteria

Of every routinely H.E.‐stained slide of 5 µm thickness, each high‐power‐field (HPF) was examined consecutively at 400× magnification as described previously by Weber et al.^24^ for all of the above mentioned pre‐defined items. The obtained results were assigned to a layer (papillary dermis, superficial, or medium reticular dermis, deep reticular dermis and subcutaneous layer).

Leukocytoclasia as reported by Dincy et al.^3^ and Mehregan et al.^4^ was noted as being present when nuclear debris in any amount was visible; erythrocyte extravasation meant extravasated erythrocytes in the proximity of 53 µm (which equals the diameter of 3.5 neutrophilic granulocytes) to a vessel. Erythrocytes due to artificial damage were excluded. For fibrin deposits, any amount of net‐like looking or occluding eosinophilic material inside of vessels was counted. Intravascular granulocytes meant all intraluminal cells and those inside of the vessel wall lining. Endothelial cell swelling was noted when more than half of the endothelial cell nuclei seemed to protrude markedly to the center of the vessel and/or were intensely enlarged and pale. Ectatic vessels included vessels with a diameter bigger than approximately 45 µm, which equals the diameter of 3 neutrophilic granulocytes. Blurred vessel borders were noted when more than half of the vessel contour was hazy. Superficial dermal edema meant the occurrence of visible clefts and pallor of the top dermal layer. Neutrophils and macrophages were additionally stained with MPO and CD163 and the immunopositive area was determined with Fiji software. Mast cells stained by Toluidine blue were counted in each HPF. All slides were evaluated by V.P. who was blinded to the prior routine histopathologic diagnosis and any clinical patient data.

### 2.5 (Immuno)histochemistry

Immunohistochemical stainings with CD163 for macrophages (1:400 overnight 4° C, monoclonal rabbit anti‐CD163 [C‐terminal], Abcam, ab189915, EPR14643‐36, Cambridge, UK) and myeloperoxidase (MPO) for neutrophils (1:400 overnight 4° C, monoclonal mouse‐anti‐human‐myeloperoxidase antibody, R&D Systems, Inc., MAB3174, 392,105, Minneapolis, USA) as well as a routine toluidine‐blue‐staining for mast cell numbers (0.5% aqueous toluidine blue solution for 24 h) were performed from paraffin‐embedded lesional skin samples (MPO: UV = 36, CSU = 27; CD163: UV = 35, CSU = 27; Toluidine blue: UV = 20, CSU = 18), each section cut at 5 µm. An immunohistochemistry protocol with Polymer‐labelled secondary antibodies (anti‐mouse: EnVision+/HRP mouse, Dako, K400011‐2, Glostrup, Denkmark; anti‐rabbit: EnVision+/HRP rabbit, Dako, K4010, Glostrup, Denmark; both applied for 30 min at room temperature) and AEC + ‐substrate system (Dako, K346111‐2, Glostrup, Denmark, applied for 10 min at room temperature) was used for detecting the primary antibodies. For the immunohistological stainings, photos were taken by an Axioplan‐II‐microscope (Zeiss) at 200× magnification and % of positivity of stained area was assessed by Fiji software.

### 2.6 Statistical analysis

Statistical analysis was performed by SPSS 24, using Mann–Whitney‐U‐Test and Chi‐square‐test (leukocytoclasia and edema, as those were categorical criteria). Fibrin inside of vessels and mast cell numbers showed a Gaussian distribution (identified by Kolmogorov‐Smirnov‐Test), for those criteria a Welch‐Test was performed additionally as statistical variances were not equal. Statistical significance was considered if *P* < 0.004 due to multiple testing according to Bonferroni correction, as in total 12 criteria were evaluated. To back up our proposed histopathologic scoring system, a decision tree was built with SPSS using the R‐plugin and a Chi‐squared automatic interaction detection (CHAID) method. The CHAID decision tree develops by distinguishing the key discriminating criteria from a starting root node that includes all criteria. Thus, it enables the analysis of interactions without assuming linear associations between independent and dependent variables.

## 3 RESULTS

### 3.1 Patient characteristics

**FIGURE 1:**
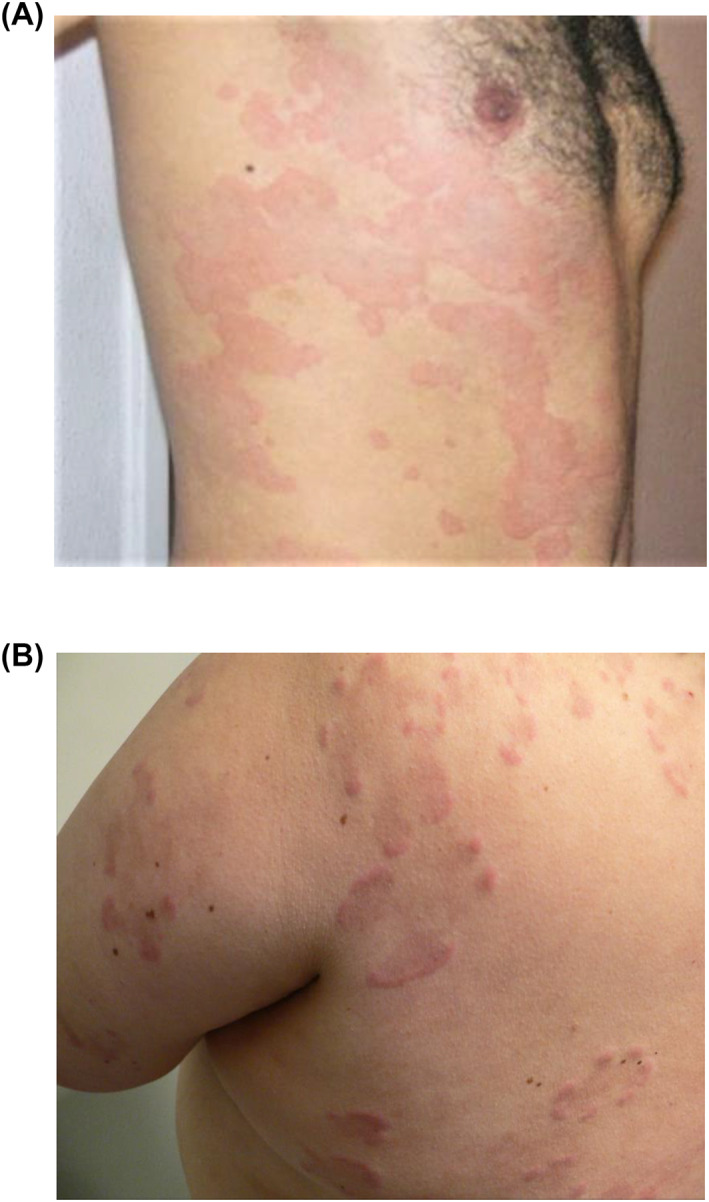
Clinical pictures of CSU and UV. (A) Urticarial lesions of a male patient with active CSU, (B) urticarial lesions of a female patient with active UV

Most demographic and clinical characteristics were similar between UV and CSU patients. Post‐inflammatory hyperpigmentation and/or a wheal duration >24 h was reported in 84.8% of UV patients and 25.5% of CSU patients. C‐reactive protein (CRP) levels were higher in most UV patients (3.6 mg/dl in UV (±8.9, *n* = 43) compared to CSU patients (0.8 mg/dl ±0.7, *n* = 51). Complement levels were normal in all available CSU patients (*n* = 36), whereas 19.4%% of the assessed UV patients (*n* = 36) showed signs of hypocomplementemia (Figure 1; Table 2).

**TABLE 2:** Demographic and clinical characteristics

Characteristic	UV	CSU
Age (years)	51.2 ± 16.0	48.2 ± 14.1
Disease duration	4.2 ± 5.6	8.2 ± 10.5
Female gender	80.4%	78.4%
Pruritus	97.8%	100%
Angioedema	69.6%	78.4%
Residual hyperpigmentation and/or wheals >24 h	84.8%	25.5%
CRP (mg/dl)	3.6 ± 8.9	0.8 ± 0.7
Hypocomplementemia (C3 < 90 mg/dl and/or C4 < 10 mg/dl)	19.4%	0%

### 3.2 Lesional UV skin, as compared to CSU, shows more leukocytoclasia, extravasated erythrocytes, fibrin deposition, endothelial cell swelling, blood vessels with blurred borders and MPO reactivity

**FIGURE 2:**
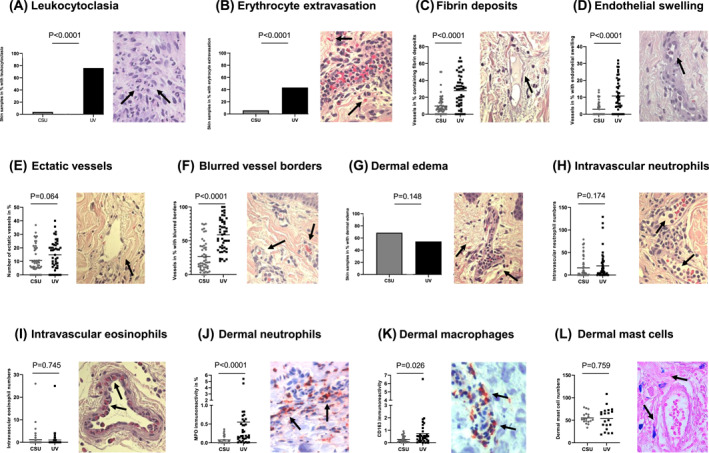
Blinded assessment of 12 predefined histopathologic criteria on slides from patients with defined clinical characteristics of either UV (*n* = 46) or CSU (*n* = 51) and matching routine histopathology. All results refer to the superficial dermal layer as histopathologic changes were most prominent in the upper dermis. Representative images of histopathologic changes observed in UV are provided for each criterion in 400× magnification.

For the blinded assessment of lesional skin biopsies from 97 patients with UV or CSU we used a total of 12 predefined histopathologic criteria: Leukocytoclasia was detected in the majority of UV patients (76%), but only in 3.9% of CSU patients (*p* < 0.0001). On average, 61.8% (±42.1%) of high power fields in biopsies of UV patients showed leukocytoclasia versus 0.9% (±5%) of CSU (*p* < 0.0001; Figure 2A). Extravasated erythrocytes were found in 41.3% of UV skin sections as compared to 2% for CSU (*p* < 0.0001; Figure 2B), corresponding to 5.2% (±8.2%) of affected vessels in UV versus 0.2% (±1.2%) in CSU (*p* < 0.0001). Intravascular fibrin deposits were found in 27.9% (±19.1%) of UV vessels compared to 9.7% (±11.2%) of CSU vessels (*p* < 0.0001; Figure 2C).

In contrast to these significant differences, other criteria did not prove as a reliable tool to distinguish UV and CSU. Endothelial cell swelling was seen in 76.1% and 43.1% of UV and CSU biopsies, respectively. On average, 10.8% (±9.7%) of vessels in UV showed endothelial cell swelling as compared to 2.9% (±4%) in CSU (*p* < 0.0001; Figure 2D). The prevalence of ectatic vessels was not significantly different (UV: 14.8 ± 10.7% of vessels vs. CSU: 10.7 ± 9.8%), and at least one ectatic vessel was found in 82.6% of UV and 78.4% of CSU samples (Figure 2E). All UV and 94.1% of CSU patients had at least one vessel with blurred borders in the superficial dermis, with 58.8% (±23.4%) and 26.5% (±22.2%) of vessels affected in UV and CSU patients, respectively (*p* < 0.0001; Figure 2F). Superficial dermal edema was seen in 54.3% of UV patients and in 68.6% of CSU patients (*p* = 0.148; Figure 2G). Intravascular neutrophil and eosinophil numbers did not differ between the groups (Figure 2H,I) as well as macrophage (CD163) and mast cell numbers (toluidine blue) (Figure 2J,K). MPO immunoreactivity, a marker for infiltrating dermal neutrophils, was higher (0.6 ± 1.1%) in UV than CSU (0.1 ± 0.1%; *p* < 0.0001; Figure 2L).

### 3.3 The urticarial vasculitis score (UVS), combining leukocytoclasia, fibrin deposition, and extravasated erythrocytes, distinguishes UV from CSU

Based on statistical significance, variability and suitability for objective assessment, we selected leukocytoclasia, intravascular fibrin deposits and erythrocyte extravasation as key criteria for diagnosing UV and developed a diagnostic score that combines their use, the urticarial vasculitis score (UVS). Leukocytoclasia, which was absent in most CSU samples, was defined as a categorical variable (yes: three points; no: 0 points). Next, we defined categories for the frequency of intravascular fibrin, that is 0‐3 points for ≥2 positive vessels in 0, 1–2, 3–4, and >5 HPFs, respectively, based on an average of 3.7 HPFs (±3.1) in UV samples with two or more positive vessels (= 2 points). Finally, we defined erythrocyte extravasation as a categorical variable and assigned two points for its presence and 0 points for its absence. Based on its subscore values, the UVS total score has a maximum of 8 and a minimum of 0 points, if the width of the section equals 5 HPFs (Table 3).

**TABLE 3:** Proposal for a urticarial vasculitis score (UVS)

	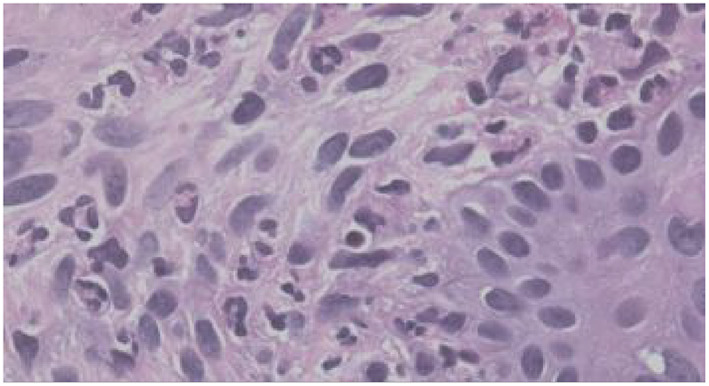	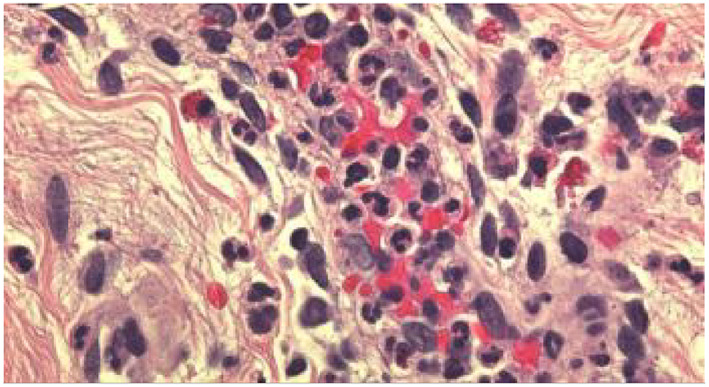	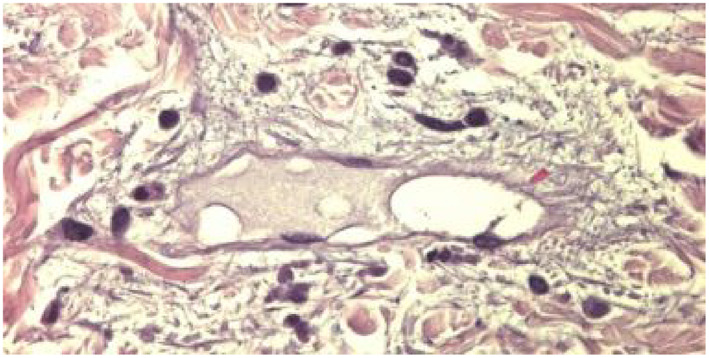
Points	Leukocytoclasia (L)	Erythrocyte extravasation (E)	Fibrin in ≥2 vessels (F)
1	‐	‐	1‐2 HPF
2	‐	If present	3‐4 HPF
3	If present	‐	5+ HPF

*Note*: W: subepidermal width in HPF.

$$\frac{5\times \left(E+L+F\right)}{W}=UVS$$

**FIGURE 3:**
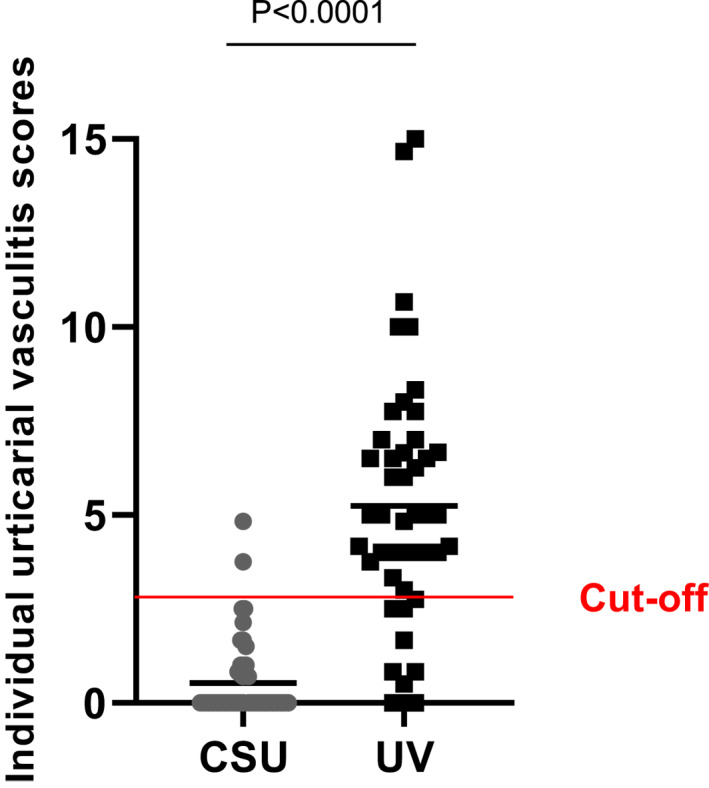
Urticarial vasculitis score (UVS): The graph shows individual UVS values for UV and CSU samples. Cut‐off 2.75

When we applied the UVS, UV samples scored on average with 5.24 points (±3.35) as compared to 0.54 points (±1.03) for CSU (*p* < 0.0001). ROC analyses identified 2.75 as a suitable UVS cut‐off for identifying UV. Of 46 patients with UV, 37 (80.4%) scored higher than 2.75 as compared to only 2 of 51 CSU patients (3.9%; Figure 3).

There were no statistically significant differences in any of the histologic parameters assessed between hypocomplementemic and normocomplementemic UV patients. However, patients with hypocomplementemia showed slightly higher UVS scores (7.9 ± 4.3 vs. 5.2 ± 3.4 points, *P* = 0.154).

### 3.4 The validity of the UVS is supported by decision tree‐based discrimination of UV and CSU

**FIGURE 4:**
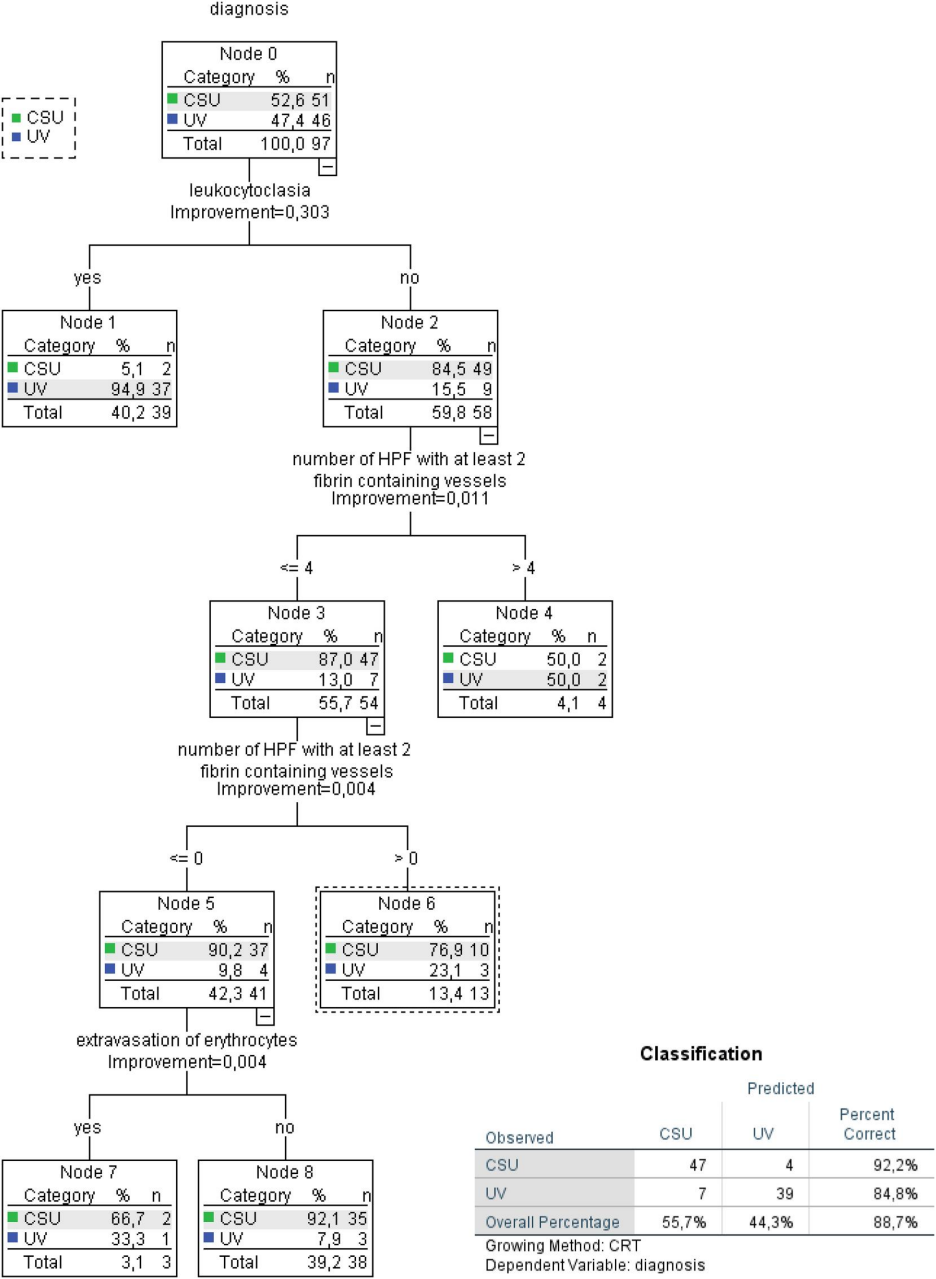
Decision tree CHAID: The first node, leukocytoclasia, shows—if it exists—that 94.9% of positive samples belong to the UV group. If a sample shows no leukocytoclasia, but in more than 4 HPF at least 2 vessels contain fibrin, there is a 50% probability that this biopsy belongs to a patient with UV. If a sample demonstrates no leukocytoclasia, no HPF with at least 2 fibrin containing vessels and no erythrocyte extravasation, the probability to belong to the CSU group is 92.1%

To assess the validity of the UVS, we used a decision tree‐based Chi‐squared automatic interaction detection (CHAID) approach (Figure 4). Using the UVS criteria, that is leukocytoclasia, fibrin deposition, and erythrocyte extravasation (with order = hierarchy), this approach diagnosed 88.7% of cases correctly, with slightly higher and lower rates than the UVS for UV (84.8%) and CSU (92.2%), respectively.

### 3.5 Use of the UVS is superior to routine histologic assessment in distinguishing UV from CSU

**FIGURE 5:**
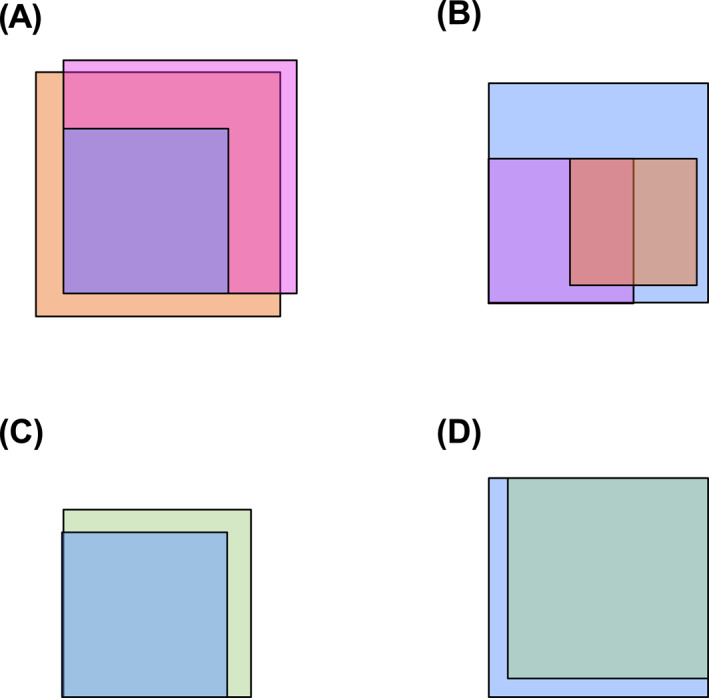
Routine histopathologic assessment of 28 randomly selected H.E.‐stained slides from lesional skin of patients with clinical and histopathologic diagnoses of UV or CSU from the Department of Dermatology and Allergy in Berlin, evaluated by three different dermatopathologists blinded to the initial diagnosis. (A) Histopathologic assessment as CSU. (B) Histopathologic assessment as UV. Blue squares (*n* = 10 assessed as CSU and *n* = 18 as UV) indicate clinicohistopathologic classification by J.R.H. Pink squares (*n* = 20 assessed as CSU and *n* = 8 as UV) indicate histopathologic classification by P.v.d.D. Orange squares (*n* = 22 assessed as CSU and *n* = 6 as UV) indicate histopathologic classification by J. C. (C) Histopathologic assessment as CSU. (D) Histopathologic assessment as UV. Blue squares (*n* = 10 assessed as CSU and *n* = 18 as UV) indicate clinicohistopathologic classification by J.R.H. Green squares (*n* = 13 assessed as CSU and *n* = 15 as UV) indicate histopathologic classification by UVS

Initially, 28 randomly selected H.E.‐stained slides from lesional skin of patients with clinical and histopathologic diagnoses of UV or CSU from the Department of Dermatology and Allergy in Berlin were evaluated by three different dermatopathologists (J.R.H., P.v.D., J.C.). In comparison, their results revealed limited agreement in assigning histopathologic findings to either UV or CSU. Combined with the knowledge of basic clinical data, more than twice as many patients were diagnosed as UV by J.R.H. (*n* = 18) compared to the histopathologic diagnoses by P.v.D. (*n* = 8) and J.C. (*n* = 6), who were both blinded to any clinical data and the previous histopathologic assessment. Only *n* = 3 slides were agreed as UV and *n* = 10 as CSU by all three dermatopathologists (Figure 5A,B).

Of these 28 samples, the UVS classified 13 as CSU and 15 as UV, missing 3 UV patients, but agreeing in all CSU patients compared to the combined clinicopathologic diagnoses by J.R.H., which means a detection rate of 100% for CSU and 83.3% for UV for this small sample (Figure 5C,D).

## 4 DISCUSSION

Until now, there is no consensus on the requirements of skin histopathology to establish a diagnosis of UV. Therefore, our study aimed at refining the histopathologic discrimination of UV from its main differential diagnosis CSU. Our results suggest that a combined assessment of three histopathologic criteria—leukocytoclasia, fibrin inside of vessels and erythrocyte extravasation—improves the assignment to either UV or CSU for the majority of cases. To our knowledge, this is the first study to develop a diagnostic score that differentiates the two groups by quantitatively evaluating a set of histopathologic criteria in UV and CSU.

The comparison of clinical parameters in patients with UV versus CSU showed no major differences, however, as described earlier,^12^, ^25^ the proportion of females was considerably higher in both groups. Of note, a quarter of all CSU patients declared either wheal duration >24 h or occasional post‐inflammatory hyperpigmentation. This may point towards an overlap or coexistence of CSU and UV. Also, the clinical phenotype may change from CSU to UV or vice versa over time. On the contrary, levels of the inflammatory marker CRP were markedly higher in UV at the time of biopsy as compared to CSU patients. Increased inflammatory markers in UV were observed before^26^ and should be followed by a diagnostic workup for underlying systemic inflammatory disorders such as lupus erythematosus. Nevertheless, minor changes in CRP have also been reported in CSU as an indication for higher severity and less response to anti‐histamine therapy, making the distinction between UV and CSU difficult based on this criterium.^27^

The histopathologic differentiation between UV and CSU is challenging and lacks standardized assessment tools. This was underlined by the limited agreement between three dermatopathologists in assigning histopathologic findings to UV or CSU. Previous studies in UV evaluated a number of histopathologic diagnostic criteria. Several authors reported leukocytoclasia and fibrin deposition as main indicators to establish a diagnosis of UV.^1^, ^2^, ^3^, ^4^, ^6^, ^10^, ^12^, ^21^, ^22^, ^23^ In line with these findings, these two criteria demonstrated the greatest differences between UV and CSU samples in our study. However, previous studies demonstrated high variability. Leukocytoclasia, for example, was found in 22.7–75% of UV patient samples,^3^, ^4^, ^5^ showing that our results (mean of 76%) are at the top end of the reported spectrum. The absence of leukocytoclasia^1^, ^2^, ^28^ in all but two of our CSU samples^6^, ^14^ matches previous findings. Interestingly, fibrin deposits were found in both, UV and CSU specimens in our study, but the number of affected vessels was significantly lower in CSU compared to UV. Former studies reported fibrin in 8.8% to 88% of UV samples,^3^, ^4^, ^5^ whereas strong fibrin deposition was seen in 1.9% of CSU patients.^29^ Erythrocyte extravasation is thought to be another common finding in UV. It was reported in 17.9–77.3% of skin specimens,^3^, ^4^, ^5^, ^29^ matching our observations (present in 41.3% of UV). Few studies in CSU described extravasated erythrocytes in up to 7.3–50% of patients,^14^, ^29^ others did not find evidence of extravasated erythrocytes.^30^ In our CSU cohort, this was a very rare finding (*n* = 1 in the superficial dermal layer).

In agreement with our study, endothelial cell swelling was noted in 76.4% to 96% of UV patients in former studies.^3^, ^5^, ^29^ Despite its higher occurrence in UV, endothelial cell swelling was observed in a considerable proportion of CSU cases in our and previous studies (1.7–80%).^29^, ^30^ This is also mirrored by the missing requirement of this criterion in the CHAID decision tree. Blurred vessel borders were mentioned in UV^5^ and less often reported for CSU.^14^ In our study it was a common finding in both diseases. To us, it does not seem to be a suitable routine diagnostic criterion as blurred vessel borders present rather as a subjective continuum with no clear cut‐off. The same accounts for dermal edema which largely depends on the investigator's perception lacking clear delineation.

Elevated neutrophil numbers in UV versus CSU skin were earlier described by staining for neutrophil elastase or MPO.^31^, ^32^ Although we found a significantly higher MPO positivity in UV patients than in CSU, it has to be acknowledged that immunohistochemical assessment of MPO was only available for the Berlin cohort. Moreover, we found a considerable variability in staining intensity and number of positive cells in this cohort suggesting that the assessment of neutrophil numbers might not serve as reliable marker for UV; additionally, the duration of wheals may as well affect the infiltrate composition and therefore MPO positivity. The number of intravascular neutrophilic granulocytes (data available from both centers) did not show significant differences between the two groups. Apart from neutrophils, other immune cells, that is, mast cells, macrophages and intravascular eosinophils, did not reveal different expression profiles in UV versus CSU.

A former study mentioned a considerable fraction of UV patients showing an inflammatory infiltrate consisting of mainly lymphocytes^5^; we did not examine this cell type in our study as this did not seem a feasible criterion for distinguishing from CSU, in which a lymphocyte‐rich infiltrate is frequently found.^2^, ^6^, ^33^

In general, it is difficult to compare the results of our study with previous reports as inclusion criteria greatly differed (e.g., no information provided about clinical presentation such as wheal duration or signs of hyperpigmentation^14^).

Limitations of our study include varying lesion sites, although lower legs were excluded because of stasis. Also, missing information about the individual wheal duration (most patients could not provide exact numbers) could have influenced the results, as the composition of the infiltrate changes over time. Another biasing factor could be the sample selection, as CSU patients with atypical clinical characteristics are more likely to get punch biopsies. As major strengths the blinded and quantitative histopathologic assessment have to be acknowledged. Confirmation of the same three histopathologic criteria by the CHAID method further supports the value of the UVS. The CHAID decision tree was earlier shown to be useful in identifying independent disease predictors and supporting medical treatment decision.^34^, ^35^ In addition, the participation of two centers—which provided similar results—enhances the validity of our findings.

In conclusion, the use of a set of predefined criteria enabled us to condense the histopathologic findings that are relevant to establish a diagnosis of UV. By quantifying the criteria leukocytoclasia, intravascular fibrin and erythrocyte extravasation we provide an easy‐to use diagnostic tool, the UVS, which may facilitate the histopathologic diagnosis of UV versus CSU. In order to evaluate the practicability and validity of the UVS, it should be applied to larger patient samples including both hypocomplementemic and normocomplementemic patients of different centers.

## CONFLICT OF INTEREST

Hanna Bonnekoh reports personal fees from Novartis, outside the submitted work. Karsten Weller reports grants and personal fees from Novartis, personal fees from Moxie, grants and personal fees from Shire/Takeda, personal fees from Biocryst, outside the submitted work. Margarida Gonçalo reports personal fees from Sanofi‐Genzyme, personal fees from Novartis, personal fees from Lilly Portugal, personal fees from Leo‐Pharma, personal fees from Pfizer, outside the submitted work. Marcus Maurer reports grants and personal fees from Allakos, grants from Amgen, personal fees from Aralez, grants and personal fees from ArgenX, grants from AstraZeneca, personal fees from Celldex, grants and personal fees from CSL Behring, grants and personal fees from FAES, grants and personal fees from Genentech, grants and personal fees from GIINNOVATION, grants from Innate Pharma, grants from Kyowa Kirin, grants from Leo Pharma, grants from Lilly, grants and personal fees from Menarini, grants and personal fees from Moxie, grants and personal fees from Novartis, grants from Roche, grants and personal fees from Sanofi/Regeneron, grants and personal fees from UCB, grants and personal fees from Uriach, grants from Centogene, personal fees from Third HarmonicBio, outside the submitted work. Karoline Krause reports grants and personal fees from Novartis, grants from Roche, personal fees from Moxie, grants from Shire/Takeda, outside the submitted work. The other authors have nothing to disclose. None declared pertaining to this work.
